# Liver myofibroblasts of murine origins express mesothelin: Identification of novel rat mesothelin splice variants*

**DOI:** 10.1371/journal.pone.0184499

**Published:** 2017-09-12

**Authors:** Michel Fausther, Elise G. Lavoie, Jonathan A. Dranoff

**Affiliations:** 1 Department of Internal Medicine, Division of Gastroenterology and Hepatology, University of Arkansas for Medical Sciences, Little Rock, Arkansas, United States of America; 2 Research Service, Central Arkansas Veterans Administration Health System, Little Rock, Arkansas, United States of America; Digestive Disease Research Center, Scott & White Healthcare, UNITED STATES

## Abstract

Liver myofibroblasts are specialized effector cells that drive hepatic fibrosis, a hallmark process of chronic liver diseases, leading to progressive scar formation and organ failure. Liver myofibroblasts are increasingly recognized as heterogeneous with regards to their origin, phenotype, and functions. For instance, liver myofibroblasts express cell markers that are universally represented such as, ItgαV and Pdgfrβ, or restricted to a given subpopulation such as, Lrat exclusively expressed in hepatic stellate cells, and Gpm6a in mesothelial cells. To study liver myofibroblasts in vitro, we have previously generated and characterized a SV40-immortalized polyclonal rat activated portal fibroblast cell line called RGF-N2 expressing multiple mesothelin mRNA transcripts. Mesothelin, a cell-surface molecule expressed in normal mesothelial cells and overexpressed in several cancers such as, mesothelioma and cholangiocarcinoma, was recently identified as a key regulator of portal myofibroblast proliferation, and fibrosis progression in the setting of chronic cholestatic liver disease. Here, we identify novel mesothelin splice variants expressed in rat activated portal fibroblasts. RGF-N2 portal fibroblast cDNA was used as template for insertion of hemagglutinin tag consensus sequence into the complete open reading frame of rat mesothelin variant coding sequences by extension PCR. Purified amplicons were subsequently cloned into an expression vector for in vitro translation and transfection in monkey COS7 fibroblasts, before characterization of fusion proteins by immunoblot and immunofluorescence. We show that rat activated portal fibroblasts, hepatic stellate cells, and cholangiocarcinoma cells express wild-type mesothelin and additional splice variants, while mouse activated hepatic stellate cells appear to only express wild-type mesothelin. Notably, rat mesothelin splice variants differ from the wild-type isoform by their protein properties and cellular distribution in transfected COS7 fibroblasts. We conclude that mesothelin is a marker of activated murine liver myofibroblasts. Mesothelin gene expression and regulation may be critical in liver myofibroblasts functions and fibrosis progression.

## Introduction

Progressive liver fibrosis, leading to cirrhosis, is the most common cause of liver failure [1]. Liver myofibroblasts are the primary effector cells during hepatic fibrosis, contributing to critical processes such as, inflammation, regeneration and remodeling [2]. In both clinical and experimental settings, liver myofibroblasts support the formation of fibrous scars observed during hepatic fibrosis. Liver myofibroblasts may derive from a variety of sources of intrahepatic origin such as, hepatic stellate cells (HSC), periportal/perivascular fibroblasts (PF), and mesothelial cells, and of extrahepatic origin such as, bone marrow-derived fibrocytes [3]. As the major fibrogenic cells driving fibrosis, liver myofibroblasts represent excellent targets for anti-fibrotic therapies. However, the specific mechanism(s) to target within liver myofibroblasts have yet to be elucidated, primarily because the signaling pathways regulating myofibroblastic activation, transdifferentiation, migration, and proliferation are still not fully understood. The explanation may partly reside in the heterogeneity of matrix-producing liver myofibroblasts [4]. Indeed, numerous recent studies using combinations of fate mapping and cell sorting methods have uncovered functional and/or phenotypic differences between liver myofibroblasts deriving from distinct (e.g.: activated HSC- vs. activated PF-derived liver myofibroblasts) [5–7] and identical (e.g.: presence or absence of αSMA expression in activated PF-derived liver myofibroblasts) [8] precursor cells. Thus, specific activation markers for these multiple liver myofibroblast (sub-)populations are still lacking, but remain critically needed.

Several laboratories including ours, have previously identified cell-surface mesothelin (Msln) as an activation marker of liver portal fibroblasts in the setting of chronic cholestasis in vivo [7,8] and, upon culture in vitro [9]. Recently, the contribution of Msln to fibrosis progression was demonstrated, as its genetic deletion in mice confers protection against experimental cholestatic liver injury [10]. Of note, the rat *Msln* gene encodes a 69-kDa preproprotein that undergoes enzymatic cleavage by a furin-like convertase to produce two mature proteins, megakaryocyte-potentiating factor (Mpf/N-Erc, 31-kDa N-terminal fragment) and Msln (C-Erc, 40-kDa C-terminal fragment) [11]. Expressed at low levels in normal mesothelial cells, both Msln and Mpf molecules are overexpressed in cancers of pleura, peritoneum, pericardium and gastrointestinal tract [12]. These distinct tumor-associated expression patterns led to suggestions of Msln and Mpf as potential biomarkers for diagnosis and prognosis of gastrointestinal cancers such as, pancreatic adenocarcinoma and cholangiocarcinoma [13–15]. Although its precise role in tumorigenesis remains poorly defined, Msln is thought to act as a malignant factor supporting metastatic progression, through regulation of key mechanisms in cancer cells such as, growth rate, resistance to cytokine-induced apoptosis, migration, adhesion, and invasiveness [16]. In addition, Msln expression is positively regulated by signaling proteins with established pro-oncogenic properties such as, TEF-1/TEAD-1 transcription factor [17] and Wnt-1 molecule [18]. Hence, distinct features such as, its cancer-specific expression, Msln deficiency in mice is associated with no overt phenotype [19], or intrinsic biological distribution, Msln is produced as cell-surface membrane-bound and -shed (soluble) forms, make Msln protein particularly attractive for the development of cancer-treating or -monitoring strategies [16]. To that effect, several Msln-targeting recombinant immunotoxins are currently tested as anti-tumor agents both in pre-clinical studies, i.e. tumor xenograft models in rodents [20], and clinical settings [11]. Altogether, these findings suggest that Msln and related pathways could be targeted to develop therapeutic approaches to disease conditions such as, fibrosis and cancer. In the present study, based on our previous observation that multiple Msln transcripts are expressed in liver myofibroblasts [9], we report the identification and characterization of novel Msln splicing variants expressed in activated rat portal fibroblasts. We also show that Msln is expressed in activated mouse hepatic stellate cells.

## Materials and methods

### Materials and reagents

Cell culture reagents and media were obtained from Life Technologies (Carlsbad, CA), Fisher Scientific (Pittsburgh, PA) and Thermo Scientific (Rockland, MA). Molecular biology and SDS-PAGE reagents/kits were obtained from Qiagen (Valencia, CA), Bio-Rad Laboratories (Hercules, CA), New England BioLabs (Ipswich, MA) and Life Technologies.

### Animal care

All procedures involving animals were reviewed and approved by University of Arkansas for Medical Sciences Institutional Animal Care and Use Committee, protocol AUP#3703. Adult male Sprague-Dawley rats (4 months, two animals) were purchased from Charles River Laboratories (Redfield, AR) and used for two-step collagenase liver perfusion performed as terminal procedure under combined ketamine (80–100 mg/kg)/xylazine (5–10 mg/kg) anesthesia (intraperitoneal administration), as previously described [9]. The method of sacrifice was exsanguination through the inferior vena cava, and all precautions taken to minimize animal suffering.

### Primary cell isolation and culture

Primary PF and HSC were isolated from rat livers, as previously described [9,21]. Briefly, hepatocyte and non-parenchymal cell fractions were obtained by *in situ* pronase/collagenase perfusion of livers. Primary PF were obtained by serial digestion and mesh filtration of hilar remnants, while primary HSC were obtained by density gradient centrifugation of non-parenchymal cell fractions. The resulting cell suspensions were plated onto tissue culture plastic dishes and grown in DMEM/F-12 containing 10% fetal bovine serum and antibiotics. Primary PF and HSC were used on day 3 or prior (quiescent, passage 0) and on day 4 and beyond (myofibroblastic, passage 0 or beyond) after plating, as previously described [9,21]. All cells were maintained at 37°C, under 95% air-5% CO2.

### Cell culture

Immortalized rat RGF, RGF-N2 activated portal fibroblasts [9], rat HSC-T6 [22], mouse Col-GFP [23], and JS1 [24] activated hepatic stellate cells, rat BDEneu cholangiocarcinoma cells [25] and COS7 fibroblasts (ATCC #CRL-1651) were grown in appropriate media supplemented with 10% fetal bovine serum and antibiotics, and maintained at 37°C, under 95% air-5% CO2, as described previously.

### RT-PCR

Total RNA was isolated from rat and mouse tissues, and primary and immortalized liver myofibroblasts cells using the RNeasy Plus Kit (Qiagen). Each RNA sample (1 μg) was digested with DNase1 enzyme (Life Technologies) to remove any genomic DNA contamination and reverse-transcribed using the iScript RT Supermix (Bio-Rad). Semi-quantitative PCR amplification was performed using RT reaction products and the TopTaq^®^ Master Mix Kit (Qiagen) with the following protocol for the PCR reactions: Initialization at 94°C for 2 minutes followed by 35 cycles of 30 second denaturation at 94°C, 30 second annealing at 60°C, 30–150 second elongation at 72°C; and 10 minutes final elongation at 72°C, using an S1000 Thermo Cycler (Bio-Rad). Amplification products were visualized on 3% agarose gels via ethidium bromide staining. The primer sequences used are listed in Table 1.

**Table 1:** Sequences of PCR primer sets used for gene expression analysis and cloning.

Identifier	Sequence	Experiment
**Rat *Msln* forward**	**GTGGTGTGAGTTGAGGGGTG**	**RT-PCR, Sequencing**
**Rat *Msln* reverse**	**GGGATGCTGTGGACAATGGA**	**RT-PCR, Sequencing**
**Rat *Msln* UTR forward**	**TGTGTCCAAACAGTGGTGTG**	**RT-PCR, Sequencing, Cloning**
**Rat *Msln* UTR reverse**	**CAGGAGCCTTAGGAGTGGTG**	**RT-PCR, Sequencing, Cloning**
**Mouse *Msln* forward**	**TGTCTCCAAACAGTGGTGTG**	**RT-PCR, Sequencing**
**Mouse *Msln* reverse**	**CAGTAGAGCTGGGACCAGGA**	**RT-PCR, Sequencing**
**Rat/Mouse *Gapdh* forward**	**TTGTGCAGTGCCAGCCTC**	**RT-PCR**
**Rat/Mouse *Gapdh* forward**	**CTGGAAGATGGTGATGGGCT**	**RT-PCR**
**Rat *Msln* OEP forward 1**	**TCTAGAATGGCCTTGCCAACAGCCCAACCCTACCCATACGATGTTCCAGATTACGCT**	**Overlap extension PCR**
**Rat *Msln* OEP forward 2**	**TACCCATACGATGTTCCAGATTACGCTCTGCTGGGGTCCTGTGGAAGC**	**Overlap extension PCR**
**Rat *Msln* OEP reverse**	**ACCGGTTCAGCTCAGTCTTAAAGCT**	**Overlap extension PCR**
**IVT forward**	**GCGAATTAATACGACTCACTATAGGGCTTAAGTATAAGGAGGAAAAAATATGGCCTTGCCAACAGCCCAACCCTAC**	**In vitro translation PCR**
**IVT reverse**	**AAACCCCTCCGTTTAGAGAGGGGTTATGCTAGTCAGCTCAGTCTTAAAGCTGAGAG**	**In vitro translation PCR**
**Gibson rat *Msln* forward**	**CCGTTTAAACTCATTACTAACCGGTTCAGCTCAGTCTTAAAGCTGAGAGC**	**Cloning**
**Gibson rat *Msln* reverse**	**ACCGATCCAGCCTCCGGACTCTAGAATGGCCTTGCCAACAGCC**	**Cloning**
**M13 forward**	**GTAAAACGACGGCCAG**	**PCR, Sequencing**
**M13 reverse**	**CAGGAAACAGCTATGAC**	**PCR, Sequencing**
**CMV forward**	**CGCAAATGGGCGGTAGGCGTG**	**PCR, Sequencing**
**TKpA reverse**	**CTTCCGTGTTTCAGTTAGC**	**PCR, Sequencing**

CMV, cytomegalovirus; IVT, in vitro translation; OEP, overlap extension PCR; TKpA, thymidine kinase polyadenylation signal; UTR, untranslated region.

### DNA plasmids and in vitro translation

RGF-N2 cDNA sample was PCR-amplified using primers located in the 5’- and 3’-UTR regions of rat Mesothelin coding sequence (NCBI Nucleotide ID: NM_031658.1). All PCR reactions were performed with Phusion^®^ (New England BioLabs) or TopTaq^®^ High-Fidelity DNA polymerases for maximal elongation fidelity. Purified PCR reactions products were cloned into the pCR4^®^ expression vector (Life Technologies), using a TOPO^®^TA cloning kit (Life Technologies). Chemically-competent OneShot^®^TOP10 bacteria cells (Life Technologies) were used for superior transformation efficiency. After PCR analysis of approximately 200 obtained transformants, six clones (A, H, S, U, W, Y) were selected based upon PCR amplicon size/abundance (ranging between 300 and 2100 base pairs approximately), and analyzed by automated sequencing to confirm insert size, sequence and orientation (UAMS DNA Sequencing Core Facility). Each DNA plasmid was then used as template for 5’-end insertion of Hemagglutinin (HA) tag consensus coding sequence (-TACCCATACGATGTTCCAGATTACGCT-, 27 base pairs) into the complete open reading frame of rat Mesothelin coding sequence, by overlap PCR extension. All constructs were designed, based upon clone sequence homology, so as to insert: 1) HA peptide coding sequence between the 24^th^ and 25^th^ base pairs (bp) of rat Mesothelin consensus coding sequence (NCBI Nucleotide ID: NM_031658.1 [24–25]), resulting in a 9-amino acid insertion between the 8^th^ and 9^th^ amino acids of rat Mesothelin protein sequence (NCBI Protein ID: NP_113846 [8–9]); and 2) *Xba1* and *Age1* restriction sites at both 5’- (before ATG start codon, NM_031658.1 [1]) and 3’-ends (after TGA stop codon, NM_031658.1 [625]) of rat Mesothelin coding sequence. All PCR amplicons were purified and used for: 1) adaptor sequences addition by PCR amplification, followed by in vitro translation reactions using the cell-free PURExpress^®^ In Vitro Protein Synthesis Kit (New England BioLabs); and 2) directional cloning into the pcDNA^™^3.3 TOPO^®^TA vector using a Gibson Assembly kit (New England BioLabs), and *Xba1* and *Age1* restriction enzymes (New England BioLabs). Obtained transformants were analyzed by PCR and automated sequencing. The primer sequences used are listed in Table 1.

### DNA transfection

COS7 fibroblasts were split into T-75 cm^2^ flasks (1.5 x 10^6^/flask) on the day before transfection. A mixture (total volume 1mL) of plasmid DNA (6 μg of each HA-Mesothelin variant expression vector) and Lipofectamine 2000 (Life Technologies) or LipoJet (Signagen, Rockville, MD) transfection reagents was incubated for 5–10 minutes at room temperature and then added to cells in a stepwise fashion, according to manufacturers instructions. Transfected cells were allowed to grow for 72 hours, before testing transgene expression. In fluorescence microscopy experiments (protein co-localization), green fluorescent protein-based CellLights Beckman 2.0 reagents (Life Technologies) specifically labeling plasma membrane cell compartment were added to transfected cells the following day (24 hrs post-transfection), and incubated for additional 24 hrs. At 48 hrs post-transfection, transfected/transduced cells were trypsinized, plated onto coverslips, and further incubated for 24 hrs. Three transfections were performed independently for each construct.

### Immunofluorescence

Transfected COS7 fibroblasts (grown on coverslips) were fixed with neutral (pH = 7.2) 4% paraformaldehyde solution (diluted in 1X Phosphate-Buffered Saline, PBS) for 20 minutes, washed in 1X PBS, and further permeabilized with Triton X-100 0.1% solution (diluted in 1X PBS) for 10 minutes, all steps at room temperature. After several washes in 1X PBS, coverslips were incubated with a 7% goat serum (Life Technologies), 0.5% bovine serum albumin (Fisher Scientific) blocking solution (diluted in 1X PBS) at room temperature for 1 hour, and then, with rabbit monoclonal anti-Hemagglutinin tag antibody (clone C29F4, Cell Signaling Technologies, Danvers, MA; diluted 1:50000 in blocking solution) at 4°C overnight. After several washes in 1X PBS, coverslips were further incubated with goat Alexa647-conjugated anti-rabbit IgG antibody (Life Technologies, diluted 1:1000 in blocking solution). After several washes in 1X PBS, DAPI-supplemented Prolong Diamond anti-fade mountant was added to coverslips. Fluorescence microscopy images were acquired using a Zeiss AxioImager imaging system (Zeiss Laboratories, White Plains, NY).

### Immunoblot

In vitro translation reaction products, primary and/or immortalized liver myofibroblast cultures, and transfected COS7 fibroblasts were re-suspended and lysed in 1X Laemmli lysis buffer (Bio-Rad) supplemented with β-mercaptoethanol (Bio-Rad) and Halt^™^ Protease Inhibitor cocktail (Thermo Scientific) for 5 min while rocking, and further denatured by boiling at 95 Celsius degrees for 5 min. In vitro translation reaction products and total cell lysates were resolved by SDS-PAGE under reducing conditions, and transferred onto a polyvinylidene difluoride membrane (Immobilon/Millipore, Bedford, MA). Membranes were blocked with 5% non-fat dry milk (Bio-Rad) in 1X Tris-Buffered Saline containing 0.1% Tween-20 (Bio-Rad) (TBS-T), incubated overnight at 4 Celsius degrees with the following primary rabbit antibodies (diluted 1:1000 in 5% BSA in 1X TBS-T): monoclonal anti-HA tag (clone C29F4, Cell Signaling Technology), polyclonal anti-HA tag (Clontech, Mountain View, CA), polyclonal anti-rat Mesothelin (clone M-285, Santa Cruz Biotechnologies, Dallas, TX), polyclonal anti-rat C-ERC/Mesothelin (#306, Clontech), and polyclonal anti-mouse C-ERC/Mesothelin (#308, Clontech) antibodies. After several washes in 1X TBS-T, membranes were further incubated with appropriate goat horseradish peroxidase-conjugated anti-rabbit IgG antibodies (diluted 1:10000 in 2.5% Milk in 1X TBS-T). After several washes in 1X TBS-T, membranes were incubated with the LumiGlo Reserve chemiluminescent substrate (KPL, Gaithersburg, MD), before band detection was achieved following exposure to CL-XPosure films (Thermo Scientific).

### Nucleotide sequences

Complementary DNA sequences containing nucleic acid residues corresponding to the complete open reading frame of rat Mesothelin splice variants were identified and assembled by single-pass automated DNA sequencing. In silico analysis of predicted amino acid sequences corresponding to rat Mesothelin splice variants was performed using open-access Big-PI Predictor, Compute pI/Mw, and SignalP 4.1 Server tools from the ExPASy Bioformatics Resources portal [26], EMBOSS Stretcher (Protein Alignment) tool from the EMBL-EBI bioinformatics web and programmatic tools framework [27], and Prop 1.0 Server tool from the DTU-CBS Prediction Servers [28]. The analysis results are listed in Table 2. A multiple alignment of cloned rat Mesothelin splice variant nucleotide sequences was performed with wild-type rat Mesothelin consensus coding sequence (NM_031658.1) as reference, and nucleotide sequence identity (as percentage) determined, using open-access MView tool from the EMBL-EBI bioinformatics web and programmatic tools framework [23] (see S1 Fig).

**Table 2:** Predicted features of wild-type and rat Msln splicing variants.

Identifier	cDNA ORF sequence length (bp)	Theoretical protein M.W. (Da)^a^	Amino acid sequence homology (%)^b^	Predicted signal peptide^c^	Predicted furin cleavage site^d^	Predicted GPI anchor motif^e^
**NM_031658.1**	**1878**	**68852.53**	**100**	**Yes**	**Yes**	**Yes**
**Clone A**	**330**	**11781.83**	**17.4**	**Yes**	**No**	**Yes**
**Clone H**	**567**	**20283.85**	**30.1**	**Yes**	**No**	**Yes**
**Clone S**	**1460**	**20903.41**	**25.1**	**Yes**	**No**	**No**
**Clone U**	**1299**	**46512.31**	**66.9**	**Yes**	**Yes**	**No**
**Clone W**	**1878**	**68784.45**	**99.4**	**Yes**	**Yes**	**Yes**
**Clone Y**	**696**	**24959.39**	**37**	**Yes**	**No**	**No**

bp, base pairs; Da, Daltons; GPI, glycosylphosphatidylinositol; M.W., molecular weight; ORF, open reading frame.

^a^Compute pI/Mw;

^b^EMBOSS Stretcher;

^c^SignalP 4.1;

^d^Prop 1.0;

^e^Big-PI Predictor bioinformatics prediction tools.

## Results

Our laboratory has previously generated and characterized two activated RGF and RGF-N2 rat liver PF cell lines that express Mesothelin (Msln) [9]. Here, we cloned and characterized these previously unknown rat Msln transcript variants expressed in RGF-N2 PF cells. In parallel, we analyze established activated HSC-T6 (rat), JS1 (mouse), and Col-GFP (mouse) hepatic stellate cell lines for the expression of prospective Msln transcript variants.

**Fig 1:**
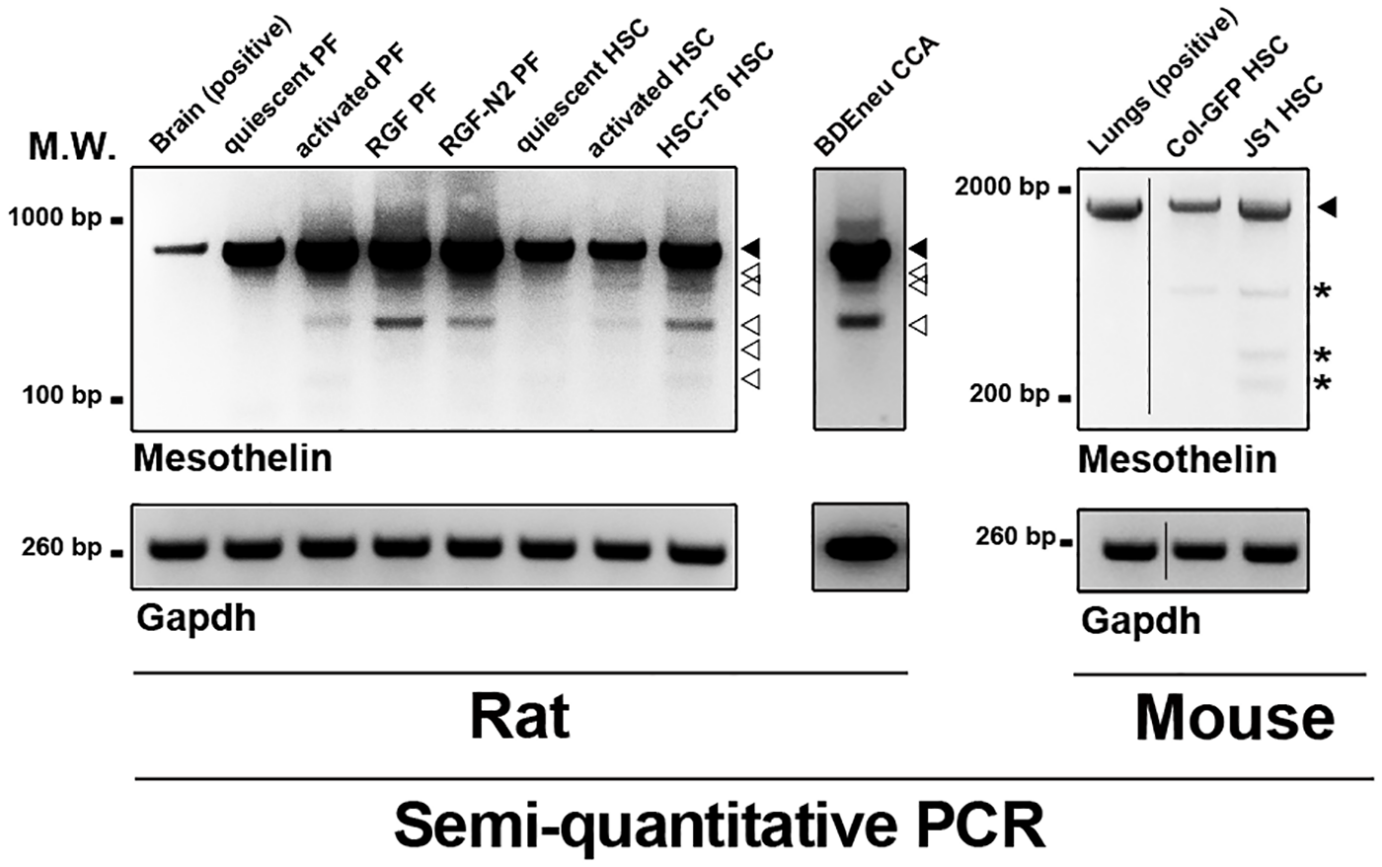
Semi-quantitative RT-PCR of Msln expression in murine liver myofibroblast and cholangiocarcinoma cells. Expression of *Msln* transcripts is analyzed in cDNA samples from primary and immortalized mouse and/or rat liver myofibroblasts, and rat cholangiocarcinoma cells. Positive controls include rat brain for rat *Msln* gene PCR, and mouse lungs for mouse *Msln* gene. *Gapdh* is used as reference. Wild-type Msln amplified is observed in all wells for both species (black arrowhead). Rat *Msln* splicing variants are also observed (empty arrowheads). Reaction artifacts were observed in mouse *Msln* PCR reactions (asterisks). Primer sequences are listed in Table 1. M.W., molecular weight; bp, base pairs; HSC, hepatic stellate cell; CCA, cholangiocarcinoma; PF, portal fibroblast.

**Fig 2:**
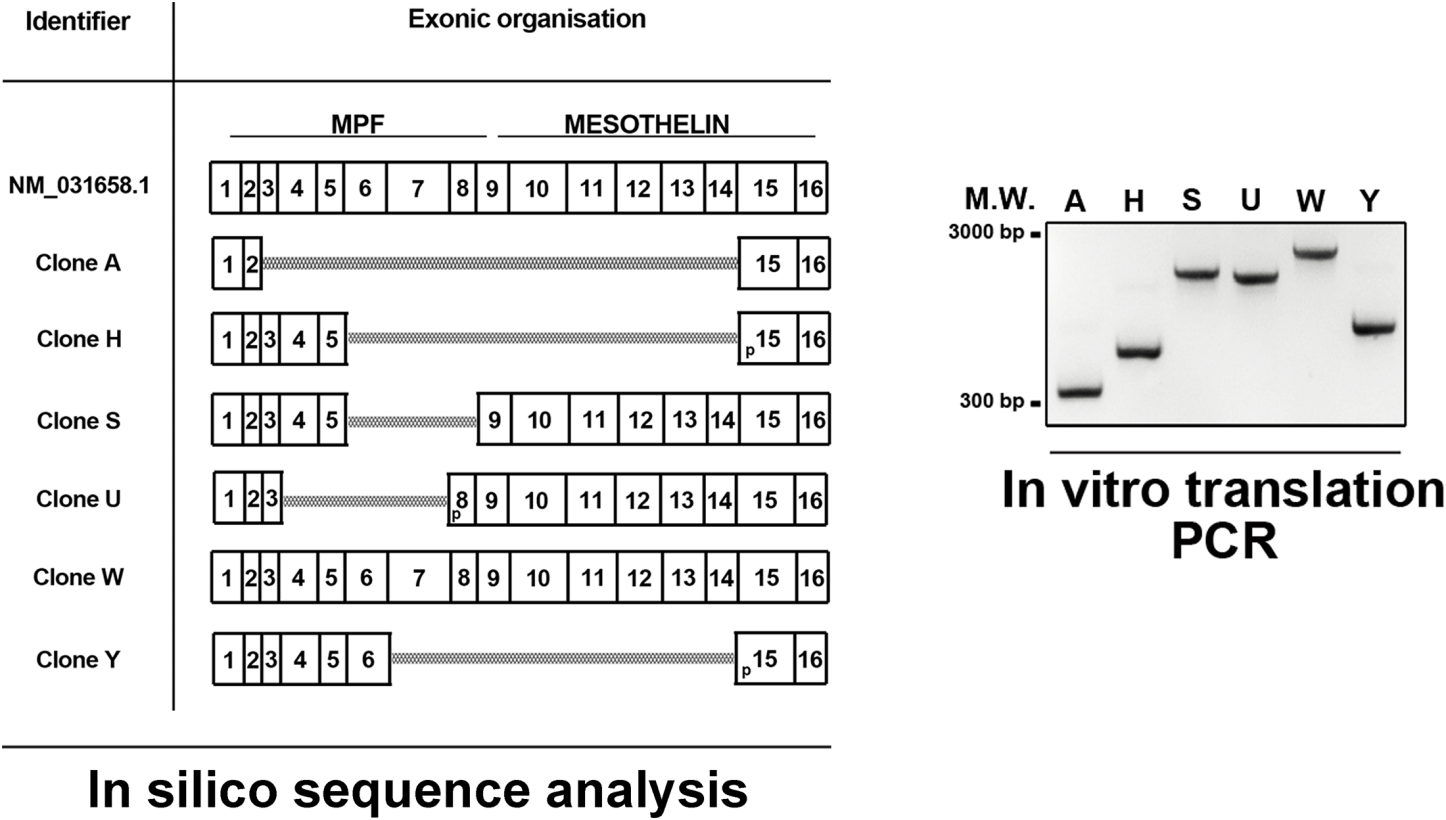
In silico analysis of *Msln* splicing variant sequences and overlap extension PCR. **LEFT PANEL**. Predicted exonic organization of obtained cDNA sequences were analyzed in silico, using wild-type *Msln* (NM_031658.1, 16 exons total) as reference. MPF, megakaryocyte potentiating factor; p, partial exon; scale bar, 100 base pairs. **RIGHT PANEL**. RGF-N2 cDNA was used as template for successive PCR amplifications with primer sets located in *Msln* UTR regions, introducing in-frame HA tag consensus sequence, and in vitro translation adaptor sequences. Each well (A, H, S, U, W, and Y) represents an individual clone corresponding to a unique *Msln* splicing variant, with molecular weights ranging between 300 to 2100 bp circa. Clone W corresponds to wild-type *Msln*. M.W., molecular weight; bp, base pairs.

First, semi-quantitative PCR analysis of cDNA samples from primary rat quiescent and activated portal fibroblasts and hepatic stellate cells ubiquitously shows an amplification product corresponding to wild-type Msln (observed molecular weight 857 base pairs, bp) (Fig 1, left panel). Interestingly, additional PCR amplification products (observed molecular weights varying between 300–800 bp) were detected only in cDNA samples from primary rat activated portal fibroblasts and hepatic stellate cells. PCR analysis was also performed using cDNA samples from immortalized rat activated RGF and RGF-N2 portal fibroblast and HSC-T6 hepatic stellate cell lines, and several amplification products, including one corresponding to wild-type Msln, were also observed. A similar observation was made for rat cholangiocarcinoma BDEneu cell line, used as PCR positive control. When semi-quantitative PCR analysis was performed on immortalized mouse Col-GFP and JS1 HSC cells, only a single amplification product corresponding to wild-type Msln was detected (Fig 1, middle panel), similar to mouse lungs PCR positive control. For each species, sequence verification of purified PCR bands of interest showed that all samples contained cDNA corresponding to Msln gene products (S1 Fig). Second, to further investigate the prospective rat Msln transcript variants, RGF-N2 cDNA was amplified by PCR, using specific oligonucleotide primers located in 5’ and 3’ untranslated regions of rat Msln mRNA sequence (NM_031658.1) i.e. capable of amplifying the complete rat Msln coding sequence (see Table 1). The resulting PCR amplification products were column-purified and used as templates for 5’-end insertion of Hemagglutinin (HA) tag consensus coding sequence by overlap extension PCR, before cloning into a CMV-driven expression vector. Based on insert size and abundance, six clones (A, H, S, U, W, and Y) were selected and sequence-verified to ensure all clones could be translated using the same open reading frame. Sequence analysis of obtained cDNAs clearly identified clone W as wild-type Msln, while the remaining clones sequences reveal mRNA exon skipping and alternative splice donor site as splicing mechanisms (Fig 2). In silico analysis predicted that encoded unmodified (i.e. HA tag-less) proteins would exhibit molecular weights ranging from 12 to 70 kiloDaltons (see Table 2). From these results, we concluded that multiple rat Msln mRNA transcripts, in addition to the wild-type isoform are expressed by liver myofibroblasts deriving from activated portal fibroblasts and hepatic stellate cells. In contrast, only wild-type Msln is expressed in mouse HSC-derived liver myofibroblasts.

**Fig 3:**
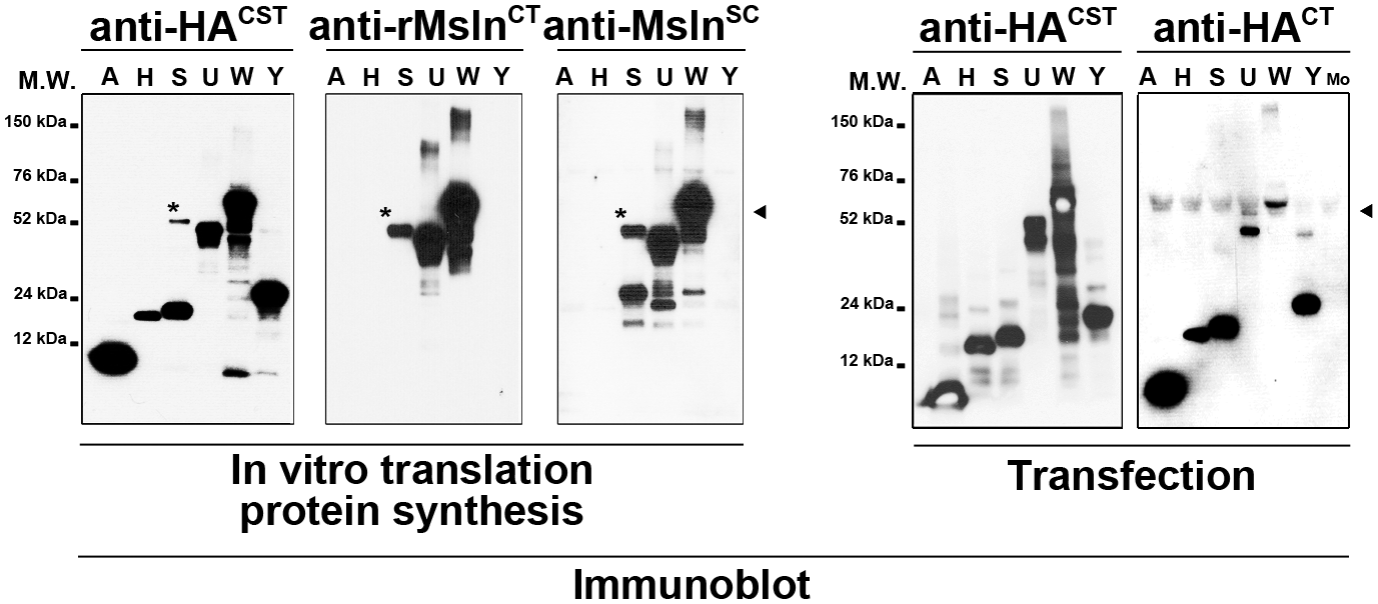
Immunoblot analysis of synthetic and recombinant *Msln* splicing variants. **LEFT PANEL**. Synthesized proteins after in vitro translation reactions using plasmid DNAs encoding Msln splicing variants A, H, S, U, W, and Y were immunoblotted with anti-HA^CST^, anti-rMsln^CT^ and anti-Msln^SC^ antibodies. In contrast to both anti-Msln antibodies, the anti-HA antibody detects bands in each well, with their molecular weight ranging from 12 to 76 kiloDaltons circa. Clone W corresponding to wild-type Msln is detected by all antibodies (black arrowhead). A reaction artifact is observed in the wells corresponding to Clone S (asterisk). **RIGHT PANEL**. Proteins samples from Msln-deficient COS7 cells transiently transfected with plasmid DNAs encoding Msln splicing variants A, H, S, U, W, and Y were immunoblotted with anti-HA^CST^ and anti-HA^CT^ antibodies. Both antibodies detect all recombinant Msln splicing isoforms. Clone W corresponding to wild-type Msln is detected by both antibodies (black arrowhead). No band is seen in the sample corresponding to mock-transfected cells (Mo). M.W., molecular weight; kDa, kiloDaltons.

**Fig 4:**
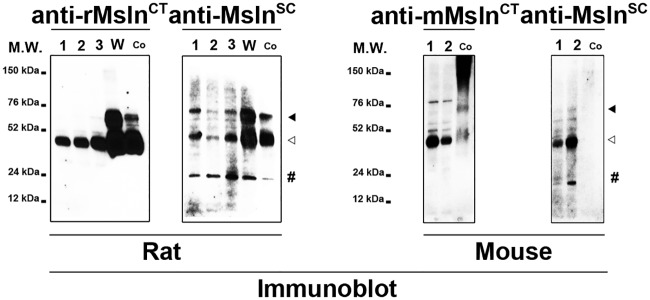
Immunoblot analysis of Msln expression in murine liver myofibroblasts. **LEFT PANEL**. Protein extracts from immortalized rat activated liver RGF (1), and RGF-N2 (2) portal fibroblasts, rat activated liver HSC-T6 hepatic stellate cells (3), COS7 cells transfected with clone W plasmid DNA (W), and COS7 cells transfected with full-length rat Msln ORF cDNA clone (Co, used as positive control) were immunoblotted with anti-rMsln^CT^ and anti-Msln^SC^ antibodies. The anti-rMsln^CT^ antibody detects the rat Msln mature form (empty arrowhead, approximately 40 kiloDaltons) in all samples, and the uncleaved form (black arrowhead, slightly under 76 kiloDaltons) in both transfected COS7 samples. The anti-Msln^SC^ antibody detects rat Msln uncleaved and mature forms. An additional band (pound, approximately 20–30 kiloDaltons), purportedly Mpf, is also observed in all wells. **RIGHT PANEL**. Protein extracts from immortalized mouse activated Col-GFP and JS1 hepatic stellate cells, and 293T cells transfected with full-length mouse Msln ORF cDNA clone (Co, used as positive control) were immunoblotted with anti-mMsln^CT^ and anti-Msln^SC^ antibodies. The anti-mMsln^CT^ antibody detects both mouse Msln uncleaved (black arrowhead, approximately 76 kiloDaltons) and mature (empty arrowhead, approximately 40 kiloDaltons) forms in all samples, while the anti-Msln^SC^ antibody only recognizes the mature form. The anti-Msln^SC^ antibody also identifies an additional band (pound, approximately 20–30 kiloDaltons), purportedly Mpf, can be seen in the JS1 sample, but does not recognize the positive control sample. M.W., molecular weight; kDa, kiloDaltons.

Subsequently, each Msln isoform clone plasmid DNA was used as template for addition of in vitro translation adaptor sequences by PCR (Fig 2). In vitro translation (IVT) reactions were performed using purified PCR products as template for recombinant protein synthesis, and analyzed by immunoblot using antibodies directed against HA tag peptide and rat Msln protein (Fig 3). As expected, the anti-HA antibody detected synthetized fusion protein products in all wells with their molecular weight varying from under 12 to slightly less than 76 kDa approximately, demonstrating that tag was successfully added and that the selected six clones encode viable recombinant proteins. The anti-rat Msln^CT^ antibody detected synthetized protein products in 3 out of 6 wells, corresponding to clones S, U and W, with molecular weights varying from under 52 to slightly less than 76 kDa approximately. Similarly, the anti-Msln^SC^ antibody detected only synthesized fusion protein products in 3 out of 6 wells, with molecular weights varying from under 24 to slightly less than 76 kDa approximately. Of note, an extra band with a molecular weight of 52 kDa was unexpectedly observed after immunoblot analysis of IVT reaction, using clone S plasmid DNA as template with anti-HA and both anti-rat Msln antibodies (Fig 3, asterisk). Because both anti-HA and anti-Msln^SC^ antibodies detected the Msln splicing isoform with a predicted molecular weight of 24 kDa (in contrast with the anti-rat (specific) Msln^CT^ antibody), this extra band was surmised to represent a reaction artifact potentially resulting from splicing isoform aggregation. Next, monkey COS7 fibroblast cell line that lacks HA tag expression was used for transient heterologous expression of HA-tagged Msln splicing variants. Immunoblot analysis of transfected cell extracts with multiple anti-HA antibodies confirmed our results obtained after similar analysis of IVT reaction products. The molecular weight of fusion protein products ranged from under 12 to 76 kDa approximately. Remarkably, only a single recombinant protein product with a molecular weight of 24 kDa was observed in the sample corresponding to S-Msln transfected cells. No positive band was detected in the sample corresponding to the mock-transfected cells. Afterwards, protein extracts from rat RGF and RGF-N2 PF, HSC-T6 HSC cell lines, and cell lysates from COS7 transfected with W-Msln plasmid DNA or a commercially-available validated full-length rat Msln cDNA clone were analyzed by immunoblot with anti-rat Msln^CT^ and anti-Msln^SC^ antibodies to assess expression of Msln protein species (Fig 4). In RGF, RGF-N2 and HSC-T6 protein samples, both antibodies only detected the mature form of Msln with a molecular weight of 52 kDa. In contrast, in COS7 transfected with W-Msln plasmid DNA and commercial rat Msln cDNA clone, both antibodies mainly detected both precursor (Mpf + Msln) and mature (Msln) forms of rat Msln, with molecular weights of 52 and 76 kDa respectively. The anti-Msln^SC^ antibody also detected in all rat samples tested a band of low molecular weight close to 20–30 kDa that could represent Mpf peptide cleaved off the Msln precursor during protein maturation. Immunoblot analysis of protein extracts from mouse Col-GFP and JS1 HSC cell lines, and control mouse Msln-transfected 293T cell lysate with anti-mouse (specific) Msln^CT^ and anti-Msln^SC^ antibodies indicated that mouse Msln mature form is expressed in both cell lines. The anti-Msln^SC^ also detected a band of low molecular weight close to 20–30 kDa in JS1 samples, similar to the observation made for rat cell line samples. However, this antibody did not produce a signal in the well corresponding to the positive control. Taken as a whole, these results clearly indicate that wild-type Msln protein is expressed in murine liver myofibroblast lines deriving from portal fibroblasts (rat species) and hepatic stellate cells (rat and mouse species).

**Fig 5:**
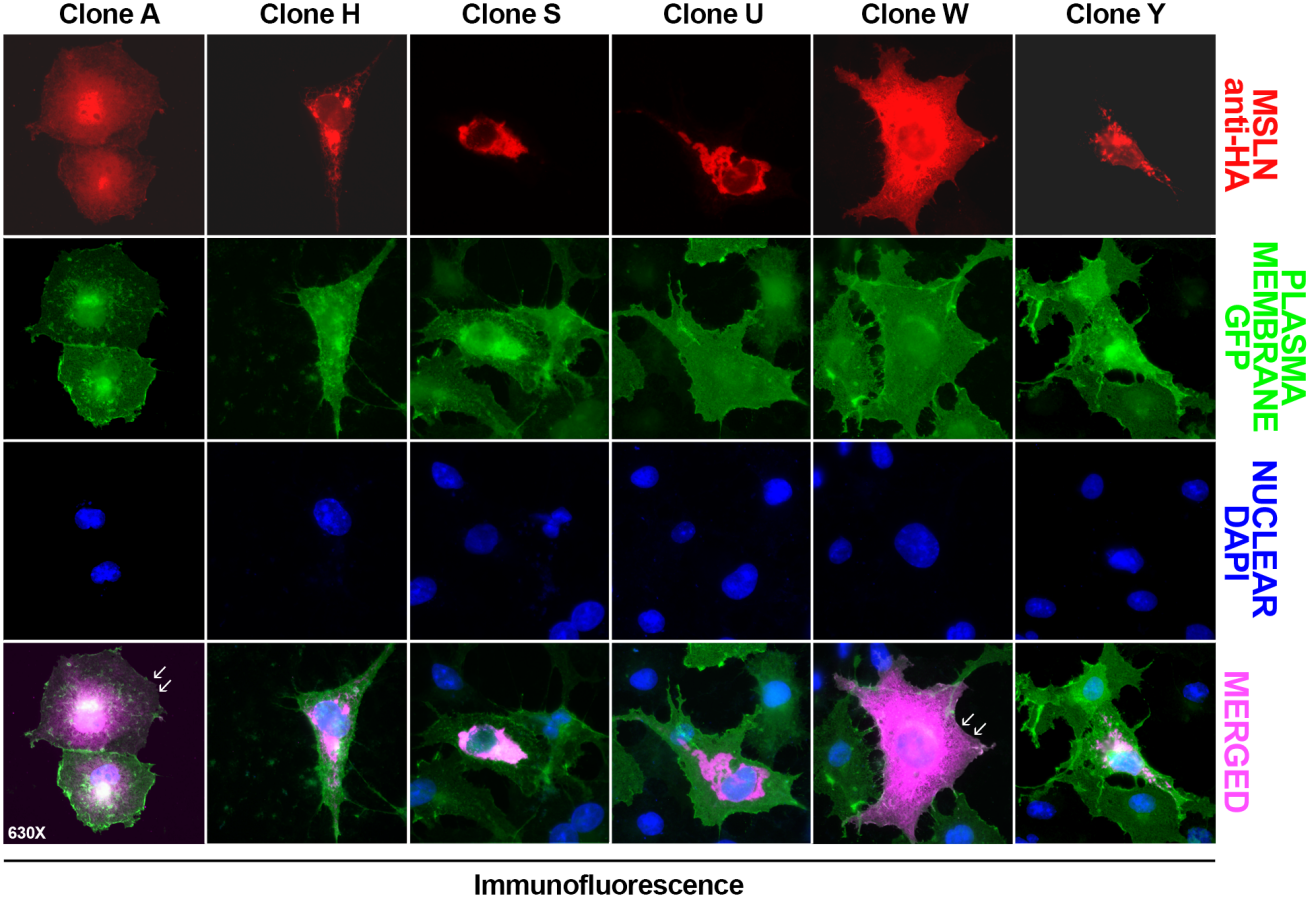
Immunofluorescence analysis of Msln splicing variant distribution in transfected COS7 cells. COS7 cells transfected with plasmid DNAs encoding Msln splicing variants A, H, S, U, W, and Y (red) were transduced to express plasma membrane-bound GFP (green), and analyzed by microscopy. While recombinant Msln A and W variants are associated with the plasma membrane (white arrowheads) and cytoplasm compartments, the remaining Msln are observed predominantly in the cytoplasmic/perinuclear area of the cells (purple). Nuclear stain is DAPI (blue). *Magnification 630X*.

Finally, the distribution of HA-tagged Msln splicing variants was monitored by immunofluorescence, upon in transfected COS7 cells expressing recombinant GFP at the level of plasma membrane (Fig 5). Of three A, H and W Msln variants predicted to possess a GPI anchor (see Table 2), only clone A and W were observed exhibiting plasma membrane localization (Fig 5). Surprisingly, the distribution of Msln isoform H appeared to be predominantly cytoplasmic/perinuclear. A similar distribution was also described for S, U and Y Msln variants lacking GPI anchorage.

## Discussion

In the present study, we show that liver myofibroblasts deriving from both rat activated portal fibroblasts (primary, immortalized RGF and RGF-N2) and hepatic stellate cells (primary, immortalized HSC-T6) express mesothelin (Msln). We also report Msln expression in liver myofibroblasts deriving from mouse (Col-GFP, and JS1) activated hepatic stellate cells. We identify novel rat Msln transcripts expressed in RGF-N2 cells distinct from the one corresponding to wild-type isoform, and encoding viable proteins that can be produced in both cell-free and cell-based expression systems. Based on sequence analysis, our study identified exon skipping and alternative donor site as differential splicing mechanisms regulating Msln gene expression in rat RGF-N2 cells. However, we need to be cautious in our assessment since these findings can arise from clonal selection bias, and the fact that relative abundance of each novel variant identified was not quantified at the mRNA level. A key point is that our cloning approach allowed us to clone these transcripts along transcripts encoding wild-type Msln isoform, which strongly argues against the notion that these are defective transcripts simply escaping nonsense mRNA-mediated decay quality control [29].

First, when the five newly identified Msln splicing variants were characterized by immunoblot with two commercially available rat Msln-specific antibodies, only three isoforms including the one corresponding to wild-type Msln could be detected at best. Our immunoblot experiments also showed that the same antibodies tested could consistently detect both recombinant uncleaved and mature forms after heterologous expression in COS7 cells, yet only one serum could recognize the native uncleaved form in rat liver myofibroblast cell lines when assayed under similar conditions. Hence, our study clearly demonstrates that available tools to track Msln gene expression products are limited in their capacity of detection. The data provided here should be useful to improve epitope mapping for generation of Msln antibodies.

Second, when the cellular distribution of Msln splicing variants was followed by immunofluorescence, one variant could be detected both at the level of plasma membrane and in the cytoplasm, similar to the wild-type protein. Other variants appear to primarily assume a cytoplasmic localization. Remarkably, cytoplasmic MSLN immunohistochemistry signals in human lung adenocarcinoma, lung squamous cell carcinoma, and extrahepatic cholangiocarcinoma tissue samples have been previously reported and attributed to likely unprocessed protein precursors [30–32]. We speculate that a potential explanation for this unexplained labeling might be the existence of yet-to-be-described human MSLN splicing variants, distinct from the other variants described in the literature [33–35]. Whether this regulatory mechanism of Msln gene expression is species-specific remains an interesting avenue that certainly needs to be further explored.

Third, our results also indicate that occurrence of Msln splicing variants can apparently be dependent on cell activation state, as rat portal fibroblasts express only wild-type Msln transcript at quiescence, with additional transcripts upon activation and phenotypic transition. A similar observation could be made for rat hepatic stellate cells undergoing myofibroblastic transdifferentiation. What could be the impact of Msln splicing variants with regards to liver myofibroblast functions? Recently, Msln, through its interaction with partner surface Mucin 16/CA125 protein, was shown to regulate proliferation, activation, and migration signals in cholestasis-induced liver myofibroblasts, and to a greater extent, liver fibrosis in vivo [10]. Hence, it is well possible that the novel Msln isoforms identified here possess the ability to also regulate the same mechanisms.

Finally, rat Msln gene splicing could also be observed in BDEneu cholangiocarcinoma, which are malignant biliary epithelial (non-fibroblastic) cells. Although our study did not characterize these cholangiocarcinoma-derived transcripts per se, a logical implication is that the observed mechanism of Msln gene expression regulation seems not to be restricted to a single cell type, here liver myofibroblasts. Importantly, this potentially provides new insights on the regulation of Msln gene expression, such as, the pathophysiological conditions and/or related factors involved the observed alternative splicing mechanisms. As suggested for liver myofibroblasts, Msln variants could influence the behavior of cholangiocarcinoma cells, since both Msln and MPF are biologically potent proteins acting as malignant factors to promote directly or indirectly tumorigenesis [36].

## Conclusions

We show that alternative splicing of the rat *Msln* gene takes place in liver myofibroblasts and malignant biliary epithelial cells. Alternative splicing of rat *Msln* mRNA precursors allows these cells to create distinct protein isoforms that might be functionally relevant to disease progression in conditions such as, fibrosis and cancer.

## Supporting information

S1 Fig Multiple alignment of rat Msln wild-type and splicing variant sequences. The assembled nucleotide sequences of cloned rat *Msln* splicing variants were aligned against wild-type rat *Msln* consensus coding sequence (NM_031658.1) using MView tool (EMBL-EBI bioinformatics web), to determine nucleotide sequence identity.
(TIF)
