# Supplementary figures and images for: Liver myofibroblasts of murine origins express mesothelin: Identification of novel rat mesothelin splice variants*
Source: PLoS One. 2017 Sep 12;12(9):e0184499. doi: 10.1371/journal.pone.0184499 (PMC5595315; doi:10.1371/journal.pone.0184499)

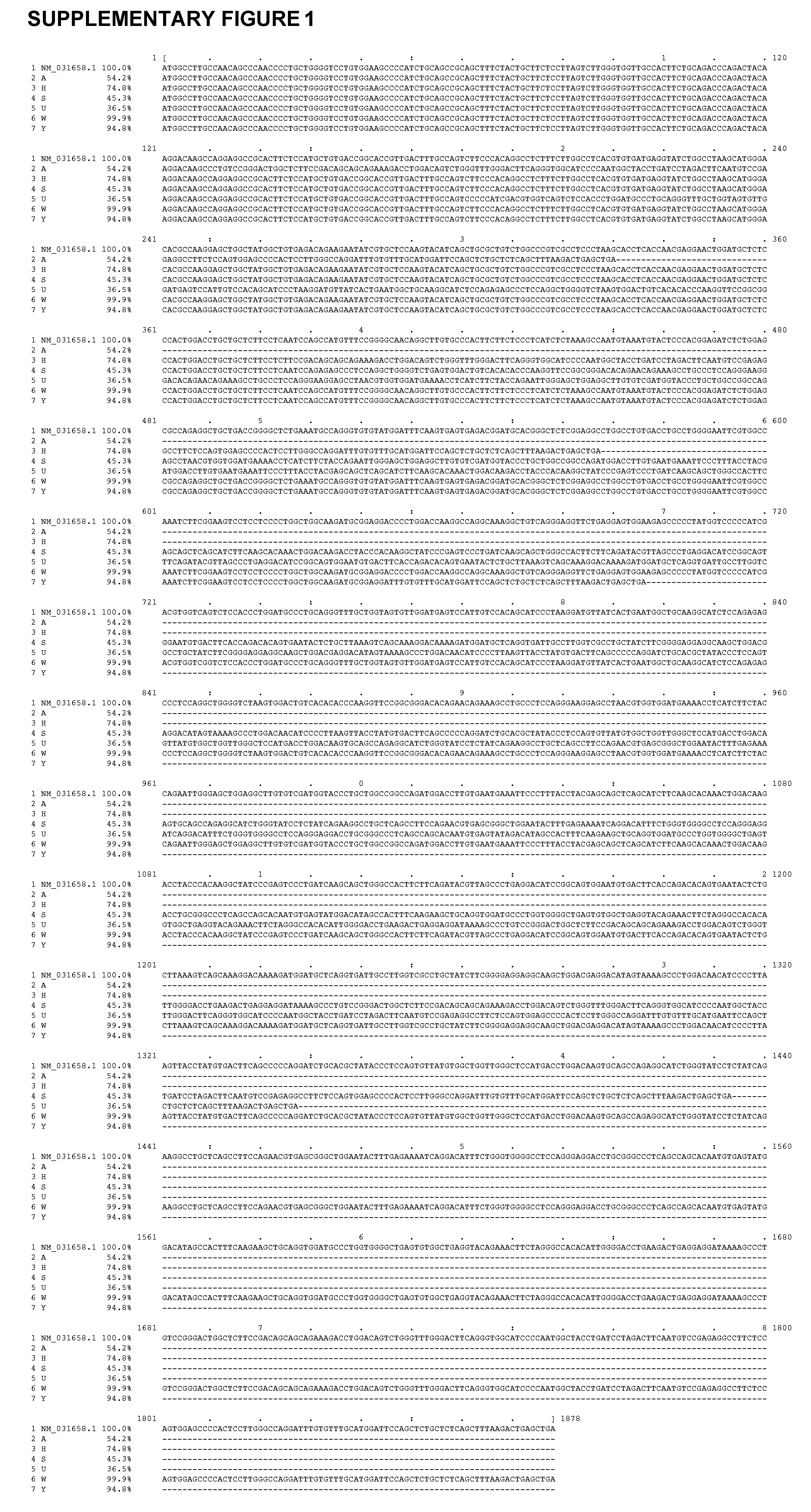

Supplement: S1 Fig — The assembled nucleotide sequences of cloned rat Msln splicing variants were aligned against wild-type rat Msln consensus coding sequence (NM_031658.1) using MView tool (EMBL-EBI bioinformatics web), to determine nucleotide sequence identity. (TIF) [file pone.0184499.s001.tif]
